# Survival After Severe COVID-19: Long-Term Outcomes of Patients Admitted to an Intensive Care Unit

**DOI:** 10.1177/08850666221092687

**Published:** 2022-04-05

**Authors:** Thanh H. Neville, Ron D. Hays, Chi-Hong Tseng, Cynthia A. Gonzalez, Lucia Chen, Ashley Hong, Myrtle Yamamoto, Laura Santoso, Alina Kung, Kristin Schwab, Steven Y Chang, Nida Qadir, Tisha Wang, Neil S. Wenger

**Affiliations:** 1 Department of Medicine, Division of Pulmonary, Critical Care, and Sleep Medicine, 12222 David Geffen School of Medicine, UCLA, Los Angeles, California, USA; 2 Department of Medicine, Division of General Internal Medicine and Health Services Research, 12222 David Geffen School of Medicine, UCLA, Los Angeles, California, USA; 3 8783 University of California, Los Angeles, California, USA; 4 Department of Medicine, Quality, 12222 David Geffen School of Medicine, UCLA, Los Angeles, California, USA; 5 Department of Medicine, 12222 David Geffen School of Medicine, UCLA, Los Angeles, California, USA

**Keywords:** COVID-19, health related quality of life, ICU

## Abstract

**Background:**

Understanding the long-term sequelae of severe COVID-19 remains limited, particularly in the United States.

**Objective:**

To examine long-term outcomes of patients who required intensive care unit (ICU) admission for severe COVID-19.

**Design, Patients, and Main Measures:**

This is a prospective cohort study of patients who had severe COVID-19 requiring an ICU admission in a two-hospital academic health system in Southern California. Patients discharged alive between 3/21/2020 and 12/31/2020 were surveyed approximately 6 months after discharge to assess health-related quality of life using Patient-Reported Outcomes Measurement Information System (PROMIS®)-29 v2.1, post-traumatic stress disorder (PTSD) and loneliness scales. A preference-based health utility score (PROPr) was estimated using 7 PROMIS domain scores. Patients were also asked their attitude about receiving aggressive ICU care.

**Key Results:**

Of 275 patients admitted to the ICU for severe COVID-19, 205 (74.5%) were discharged alive and 132 (64%, median age 59, 46% female) completed surveys a median of 182 days post-discharge. Anxiety, depression, fatigue, sleep disturbance, ability to participate in social activities, pain interference, and cognitive function were not significantly different from the U.S. general population, but physical function (44.2, SD 11.0) was worse. PROPr mean score of 0.46 (SD 0.30, range −0.02 to 0.96 [<0 is worse than dead and 1 represents perfect health]) was slightly lower than the U.S. general population, with an even distribution across the continuum. Poor PROPr was associated with chronic medical conditions and receipt of life-sustaining treatments, but not demographics or social vulnerability. PTSD was suspected in 20% and loneliness in 29% of patients. Ninety-eight percent of patients were glad they received life-saving treatment.

**Conclusion:**

Most patients who survive severe COVID-19 achieve positive outcomes, with health scores similar to the general population at 6 months post-discharge. However, there is marked heterogeneity in outcomes with a substantial minority reporting severely compromised health.

## Introduction

The coronavirus-2019 (COVID-19) pandemic, caused by the severe acute respiratory syndrome coronavirus 2 (SARS-CoV-2), has resulted in over 4 million deaths worldwide as of July 2021.^1^ While the acute phase of the illness has been well described,^2-6^ the post-discharge outcomes of patients who were critically ill from COVID-19 has not been well-reported, particularly in the United States (U.S.).^7-12^ As such, we sought to describe long-term outcomes of patients who required admission to an intensive care unit (ICU).

Survivors of acute critical illness often suffer from post-intensive care syndrome (PICS), a collection of physical, cognitive, and psychological impairments that arise from critical illness, persisting long after a patient leaves the ICU.^13-15^ PICS can contribute to decreased health-related quality of life (HRQOL).^16,17^ PICS can also lead to financial strain, with 40% of ICU survivors unable to return to work a year after hospital discharge.^18,19^ Admission to the ICU for COVID-19 involves isolation from family, uncertainty, and limited human interaction—factors that are predictive of long-term psychological harm for ICU patients and their families.^20-24^ Delirium, which is associated with persisting cognitive impairment after discharge,^25^ is common in COVID-19 patients in the ICU.^20,26^ Furthermore, infection prevention mandates make coordination of care such as inpatient rehabilitation more challenging for COVID-19 patients, placing them at a higher risk for deconditioning. Taken together, these factors might be expected to cause severe PICS for the survivors of COVID-19 ICU care, but this has not been clearly elucidated.

We aimed to characterize post-discharge HRQOL approximately 6-months after discharge for patients admitted to the ICU with severe COVID-19. We also surveyed whether patients were glad they received aggressive critical care given their current state—information that might help providers better engage future patients and families in informed decision-making regarding invasive life-sustaining interventions. With an ever-increasing number of COVID-19 survivors, it is critical to understand the trajectory of severe COVID-19 and optimize recovery after hospital admission.

## Methods

This is a prospective cohort study conducted at a two-hospital academic health system in Southern California. The study was approved by the health system's Institutional Review Board (IRB #20-000817) and is reported in accordance with the STrengthening the Reporting of OBservational studies in Epidemiology (STROBE) reporting guideline.^27^

### Participants

Patients were included in the study if they were at least 18 years old, admitted to the ICU for laboratory-confirmed COVID-19, and discharged between March 21 and December 31, 2020. Patients transferred to another acute care hospital and those who incidentally tested positive but were admitted to the ICU for unrelated reasons were excluded. Demographics, comorbidities, highest Sequential Organ Failure Assessment (SOFA)^28^ score within the first 24 hours of ICU admission, clinical course, and discharge status were retrospectively abstracted from the electronic medical record (EMR) and stored in REDCap.^29^ Elixhauser comorbidity index (ECI) – a set of comorbidities that predict in-hospital mortality – was calculated for each patient.^30^ Each patient's Social Vulnerability Index (SVI) was computed based on their address. The SVI uses 15 census variables including poverty and housing, and is associated with a community's capacity to respond and recover when confronted by external stresses on human health.^31,32^

Patients who survived to discharge were eligible for post-hospital surveys. Surveys were mailed to the patient's home two weeks prior to their 3-month and 6-month post-hospital discharge dates. For the analyses reported, we used the survey completed closest to 6-months after discharge. The mailing included an introductory letter, an informational sheet that described risks, benefits, and alternatives to participating, the survey itself, and a stamped pre-addressed return envelope. A Spanish-language version of the survey was mailed to patients for whom the EMR specified that Spanish was their preferred language. Reminder cards were sent one week later. After 2 weeks, study personnel called the patient to prompt completion, offer an online option if preferred, or conduct the survey over the telephone. Non-responders received at least two telephone calls, one after 5pm. Survey participants received a $25 gift card. For patients who did not respond to the survey, post-discharge clinical status was abstracted from the EMR including information obtained from the institution's Ambulatory Monitoring Program, a readmission prevention clinical program in which nurses call COVID-19 patients to query symptoms and whether patients have returned to baseline health.

### Survey Instruments

The survey (eSupplement 1) included Patient-Reported Outcomes Measurement Information System (PROMIS®) measures.^33^ We incorporated the PROMIS-29 v2.1, which includes a pain intensity item and 4 items assessing each of 7 domains: physical function, anxiety, depression, fatigue, sleep disturbance, social participation, and pain interference;^34^ the PROMIS Cognitive Function Short Form 8a V2.0;^35^ and four PROMIS global health questions (2 physical health and 2 mental health).^36^ PROMIS measures have undergone extensive development and evaluation in the general population and a variety of chronic diseases.^33,37-39^ PROMIS scores are reported on a T-score metric, with a mean of 50 and standard deviation of 10 in the general U.S. population. We used pattern-based item response theory scoring for the PROMIS measures.^40^ PROMIS-29 domains had excellent internal consistency reliability in this study: coefficient alpha was 0.95 for physical function, 0.96 for anxiety, 0.94 for depression, 0.96 for fatigue, 0.88 for sleep disturbance, 0.98 for social participation, 0.99 for pain interference, and 0.99 for cognitive function. We also estimated the PROMIS-29 physical and mental health summary scores.^41^ In addition, we estimated the PROMIS-Preference (PROPr) preference-based utility score from 7 PROMIS domains: depression, fatigue, pain interference, physical function, sleep disturbance, ability to participate in social activities, and cognitive function. The PROPr was calculated using SAS (version 9.4) and ranges from −0.022 to 1.0 where < 0 is worse than dead and 1.0 is perfect health in a representative U.S. sample.^42-44^ Finally, seven questions were added in month three of the study to assess current physical function, cognition, mood, fatigue, sleep, social activities, and pain compared to baseline prior to COVID-19 infection.

Post-traumatic stress disorder (PTSD) was assessed by the short form of PCL-5 (PTSD Checklist).^45,46^ The four questions are scaled 0–4 and a sum of ≥ 8 was used to indicate probable PTSD.^46^ To evaluate the potential effect of isolation, we used a 3-item Loneliness Scale, in which a score ≥ 6 indicates loneliness.^47^ These three items were derived from the revised UCLA Loneliness Scale and are a subset of another short-form measure.^48^ The survey also asked whether the patient had a paying job prior to hospitalization and whether they returned to work. Lastly, the survey asked whether the patient was glad that they received aggressive life-sustaining treatment for COVID-19 and whether they would be willing to receive this type of treatment again.

### Statistical Analysis

We characterized and compared patients who died during the hospitalization and those discharged alive. Among patients who survived to discharge, we compared survey responders and non-responders. Bivariate comparisons between patient groups were performed using χ2, Fisher's exact, two-sample t-tests, and Wilcoxon rank-sum tests as appropriate. One-sample *t*-tests were used to compare standardized PROMIS scores to a general U.S. population average of 50. All analyses were two-tailed and a p-value < 0.05 was considered statistically significant. Analyses were performed using STATA/SE 15.1 and R statistical software (version 4.1.0).

Kruskal-Wallis tests were used to test the association between PROPr and categorical variables, and Pearson's correlation coefficient for continuous variables. Baseline characteristics and outcomes were compared between patients who had PROPr > 0.2 and those with PROPr ≤ 0.2, representing patients with very low health utilities.

## Results

### Study Cohort

**Figure 1.**
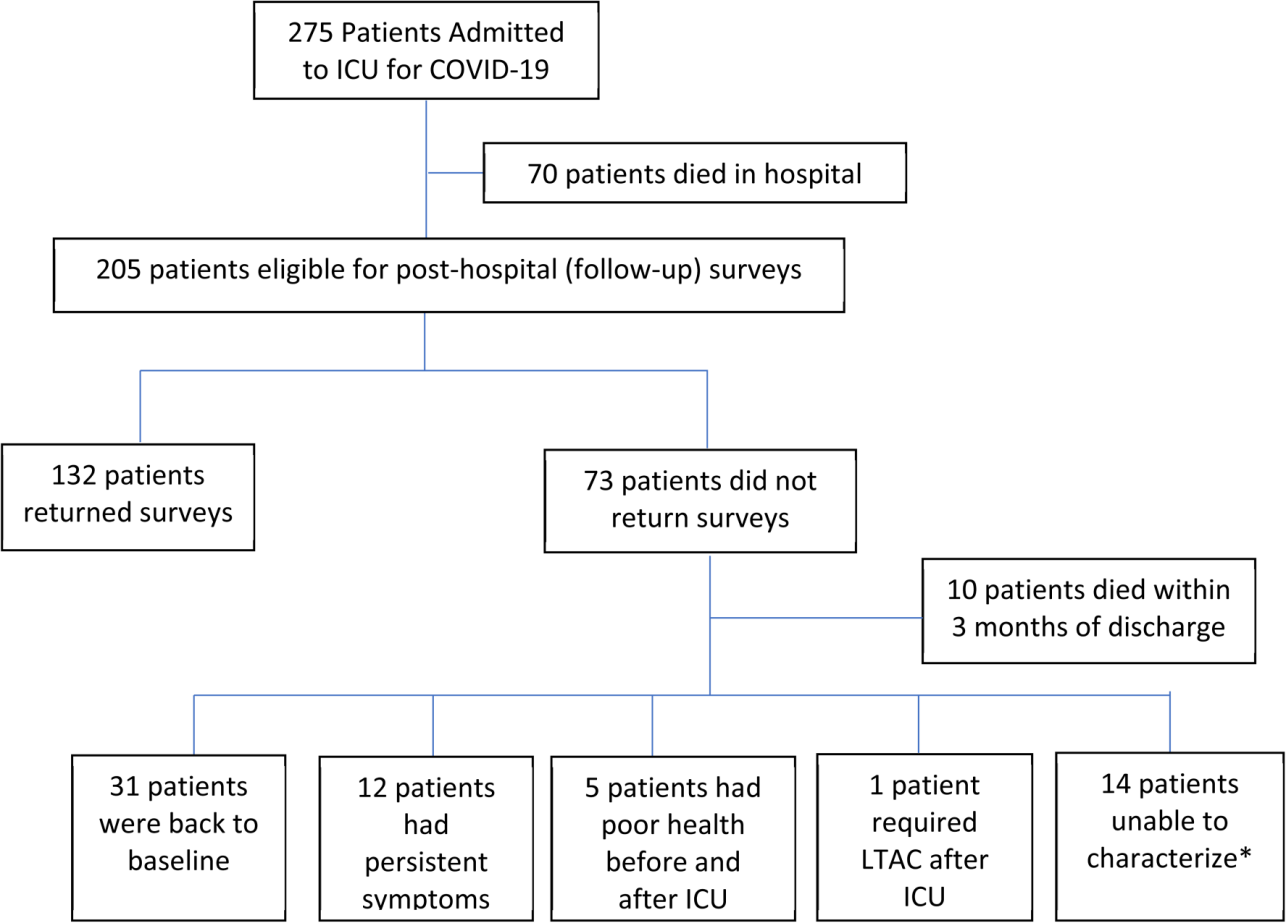
Flow diagram of study cohort.

Between March 11, 2020 (start of the pandemic in Los Angeles) and December 31, 2020, 275 patients were admitted to the health system's ICUs for COVID-19 and were discharged (n = 205) or died inpatient (n = 70) (Figure 1). Compared to those who died, the 75% of patients who survived ICU admission for severe COVID were younger, had lower ICU admission Sequential Organ Failure Assessment (SOFA) scores, lower ECI, shorter ICU stays, and were less likely to receive mechanical ventilation (p < 0.05 for all comparisons, Table 1). Survivors were more likely to have received dexamethasone (recommendations favoring dexamethasone were announced in July 2020).^49^ They were also more likely to have private insurance and have English as their primary language, although they had similar race and ethnicity and SVI to those who died.

Study cohort of all patients admitted to health system ICUs for COVID-19 and died or discharged by December 31, 2020. Status in last row determined by outpatient notes in the electronic medical record.

ICU = intensive care unit; LTAC = long term acute care

*includes 3 patients who died within 1 year of discharge.

**Table 1.** Characteristics of Study Cohort.

	Patients Admitted to ICU for COVID-19 (N = 275)		Patients Admitted to ICU for COVID-19 and Survived to Hospital Discharge (N = 205)	
Characteristics	Survived to hospital discharge (N = 205)	Died in hospital (N = 70)	Did not complete survey (N = 73)	Completed survey (N = 132)
Age (years), median (IQR)*	59.6 (48.2-70.3)	71.3 (57.1-79.6)	62.0 (49.1-74.4)	59.1 (47.5-68.8)
Female, n (%)	87 (42.4%)	26 (37.1%)	27 (37.0%)	60 (45.5%)
Race/Ethnicity, n (%)				
** **Non-Hispanic White	43 (21%)	16 (22.9%)	19 (26.0%)	24 (18.2%)
** **Hispanic	108 (52.7%)	38 (54.3%)	30 (41.1%)	78 (59.1%)
Black	16 (7.8%)	5 (7.1%)	7 (9.6%)	9 (6.8%)
** **Asian	19 (9.3%)	6 (8.6%)	8 (11.0%)	11 (8.3%)
** **Native Hawaiian or Pacific Islander	1 (0.5%)	0 (0.0%)	1 (1.4%)	0 (0.0%)
** **Other	18 (8.8%)	5 (7.1%)	8 (11.0%)	10 (7.6%)
Preferred Language, n (%)*				
** **English	140 (68.3%)	36 (51.4%)	54 (74.0%)	86 (65.2%)
** **Spanish	52 (25.4%)	22 (31.4%)	14 (19.2%)	38 (28.8%)
** **Other	13 (6.3%)	12 (17.1%)	5 (6.8%)	8 (6.1%)
Insurance, n (%)*				
** **Private	86 (42.0%)	15 (21.4%)	26 (35.6%)	60 (45.5%)
** **Public/MediCal/Medicare	104 (50.7%)	46 (65.7%)	41 (56.2%)	63 (47.7%)
** **Other/Unknown	15 (7.3%)	9 (12.9%)	6 (8.2%)	9 (6.8%)
Social Vulnerability Index, median (IQR)†	0.6 (0.3-0.8)	0.6 (0.2-0.9)	0.4 (0.2-0.7)	0.7 (0.3-0.8)
Comorbidities, n (%)				
** **Hypertension	112 (54.6%)	45 (64.3%)	41 (56.2%)	71 (53.8%)
** **Diabetes	81 (39.5%)	22 (31.4%)	30 (41.1%)	51 (38.6%)
** **Heart Failure	13 (6.3%)	5 (7.1%)	3 (4.1%)	10 (7.6%)
** **Chronic Kidney Disease†	44 (21.5%)	17 (24.3%)	22 (30.1%)	22 (16.7%)
** **Chronic Lung Disease	19 (9.3%)	9 (12.9%)	4 (5.5%)	15 (11.4%)
** **Active Cancer	4 (2.0%)	7 (10.0%)	3 (4.1%)	1 (0.8%)
Highest SOFA within 24** **hours of ICU admission, median (IQR) *	3.0 (0.0-7.0)	10.5 (4.2-13.0)	3.0 (1.0-8.0)	3.0 (0.0-5.2)
Elixhauser Comorbidity Index, median (IQR) * †	9.0 (0.0-18.0)	27.0 (13.0-33.0)	10.0 (4.0-26.0)	8.0 (0.0-16.2)
BMI, median (IQR)	28.5 (24.4-33.5)	28.2 (23.5-34.5)	28.1 (24.2-33.8)	29.0 (24.5-33.2)
Organ Transplant Recipient, n (%)	19 (9.3%)	9 (12.9%)	7 (9.6%)	13 (9.8%)
Admitted from:				
** **Emergency Department	173 (84.4%)	52 (74.3%)	64 (87.7%)	109 (82.6%)
** **Outside hospital transfer	32 (15.6%)	16 (22.9%)	9 (12.3%)	23 (17.4%)
** **Other	0 (0.0%)	2 (2.9%)	0 (0.0%)	0 (0.0%)
Hospital length of stay, median days (IQR)	13.0 (8.0-21.0)	13.0 (7.0-17.8)	13.0 (8.0-21.0)	13.0 (8.0-22.2)
ICU length of stay, median days (IQR)*	5.0 (2.0-12.0)	9.5 (4.0-15.0)	4.0 (2.0-10.0)	6.0 (2.0-13.0)
Treatment with, N (%)				
** **Remdesivir†	135 (65.9%)	42 (60.0%)	36 (49.3%)	99 (75.0%)
** **Convalescent Plasma	71 (34.6%)	26 (37.1%)	20 (27.4%)	51 (38.6%)
** **Tocilizumab	24 (11.7%)	8 (11.4%)	11 (15.1%)	13 (9.8%)
** **Sarulimab	4 (2.0%)	0 (0.0%)	0 (0.0%)	4 (3.0%)
** **Dexamethasone*	106 (51.7%)	24 (34.3%)	31 (42.5%)	75 (56.8%)
** **Hydroxychloroquine	22 (10.7%)	7 (10.0%)	11 (15.1%)	11 (8.3%)
** **Leronlimab	14 (6.8%)	5 (7.1%)	5 (6.8%)	9 (6.8%)
Utilized life sustaining treatments, n (%)				
** **CPR*	2 (1.0%)	5 (7.1%)	1 (1.4%)	1 (0.8%)
** **Mechanical Ventilation*	74 (36.1%)	57 (81.4%)	26 (35.6%)	48 (36.4%)
** **Vasopressors*	75 (36.6%)	59 (84.3%)	27 (37.0%)	48 (36.4%)
** **Dialysis*	22 (10.7%)	27 (38.6%)	13 (17.8%)	9 (6.8%)†
** **ECMO*	7 (3.4%)	8 (11.4%)	2 (2.7%)	5 (3.8%)

CPR = cardiopulmonary resuscitation, ECMO = extracorporeal membrane oxygenation, ICU = intensive care unit, IQR = interquartile range, SOFA = Sequential Organ Failure Assessment.

χ2 and Fisher's exact test were used for categorical variables. Wilcoxon rank-sum test was used for continuous variables.

*p < 0.05 when compared with patients to survived to hospital discharge.

†p < 0.05 when compared to patients who did not complete survey.

Of the 205 patients who were discharged from the ICU, 10 (4.9%) died before receiving 3-month surveys and 132 (64.3%) completed either or both 3-month and 6-month surveys. Surveys used in this analysis were completed a median of 182 (IQR 173-188) days after hospital discharge. Those who completed surveys had higher Social Vulnerability Index scores (on 0-1 scale, 0.7 vs. 0.4, p = 0.004), lower Elixhauser comorbidity index (8.0 vs. 10.0, p = 0.003), and were less likely to have chronic kidney disease (16.7% vs. 30.1%, p = 0.038) (Table 1) than those who did not return surveys. Other variables in Table 1 were not significantly different between survey responders and non-responders.

Of the 63 non-respondents who survived three months after discharge, 31 (49.2%) reported being “back to baseline” health and 12 (19.0%) reported continued symptoms according to Ambulatory Monitoring Program outpatient notes (Figure 1). Five (7.9%) were in extremely compromised health states (eg, advanced dementia in long term care) before COVID-19 infection, 1 (1.6%) became severely compromised due to COVID-19 (discharged obtunded to a long-term acute care facility), and 14 (22.2%) were unable to be characterized (3 were known dead within one year of discharge).

The surveyed sample of 132 survivors of COVID-19 in the ICU had a median age of 59 years with the majority being male, Hispanic and having English as their preferred language. Insurance was nearly evenly split between commercial and public. About half were working, 47% had a family income less than $35,000, 41% had a high school education or less, and median SVI was 0.7. Most participants had at least one co-morbid condition and nearly 10% had received an organ transplant. Mean body mass index (BMI) was 29 (overweight, nearly obese). Nearly all were treated with at least one COVID-19-specific therapy with over one-third requiring ventilator support and vasopressors during the ICU stay. The median length of stay in the ICU and the hospital was 6 days and 13 days, respectively. (Tables 1 and 2)

**Table 2.** Survey Responses of Patients who Survived ICU Admission for COVID-19 (N = 132).

Number of people in household (N = 114)	
1	8 (7.0%)
2	23 (20.2%)
3	25 (21.9%)
4	19 (16.7%)
5	21 (18.4%)
More than 5	18 (15.8%)
Income (N = 120)	
Less than $20,000	34 (28.3%)
$20,000 to $34,999	22 (18.3%)
$35,000 to $49,999	20 (16.7%)
$50,000 to $74,999	7 (5.8%)
$75,000 to $99,999	6 (5.0%)
$100,000 to $150,000	11 (9.2%)
More than $150,000	20 (16.7%)
Highest Level of Education	
Less than high school graduate	35 (27.3%)
High school graduate	18 (14.1%)
Some college	34 (26.6%)
College graduate	25 (19.5%)
Post graduate education	16 (12.5%)
Current living situation	
Home	123 (93.8%)
Assisted Living	1 (0.8%)
Skilled Nursing Home	4 (3.1%)
Other*	3 (2.2%)
Requires supplemental oxygen*	22 (16.7%)
Currently needs a caregiver*	33 (25%)
Readmitted to hospital since discharge	12 (9.2%)
Work	
Had paying job before illness	68 (52.3%)
Returned to work	40 (58.8%)
Time before returning to work, median weeks (IQR)	6.0 (3.5-13.0)
Employed to prior level	32 (47.1%)
Measure	Score
PTSD Checklist-5, mean score (SD)	3.48 (4.33)
N with score ≥ 8 (PTSD probable), (%)	26 (20.3%)
Loneliness Scale, mean score (SD)≥ 6	4.4 (2.0)
N with score ≥ 6 (likely lonely), (%)	38 (29.2%)
Are you glad that you received aggressive life-sustaining treatment for COVID-19 when in the hospital?	
No, I would rather have had comfort care even though I probably would have died	3 (2.3%)
Yes, I am glad I receive life-sustaining treatment	125 (97.7%)
If yes, would you be willing to receive this type of treatment again, if needed?	
No	6 (4.8%)
Yes	118 (95.2%)

ICU = intensive care unit, SD = standard deviation.

*One patient was living at memory care center and two were living with relatives. Two patients were receiving supplemental oxygen before COVID-19 admission. Twelve patients had a caregiver before COVID-19 admission.

### Outcomes of Patients who Survived COVID-19 ICU Care

Six months after hospital discharge, 94% of respondents were living at home, 16% required help from a caregiver that was not needed before COVID-19, and 17% were on supplemental oxygen (2 of whom were previously on oxygen). Of the 68 respondents who were employed before hospitalization, 40 (59%) were back to work, with a median hiatus of 6 weeks, and 32 (47%) employed at their pre-morbid level. Twelve (9%) had been readmitted to the hospital. (Table 2)

### HRQOL Outcomes

**Figure 2.**
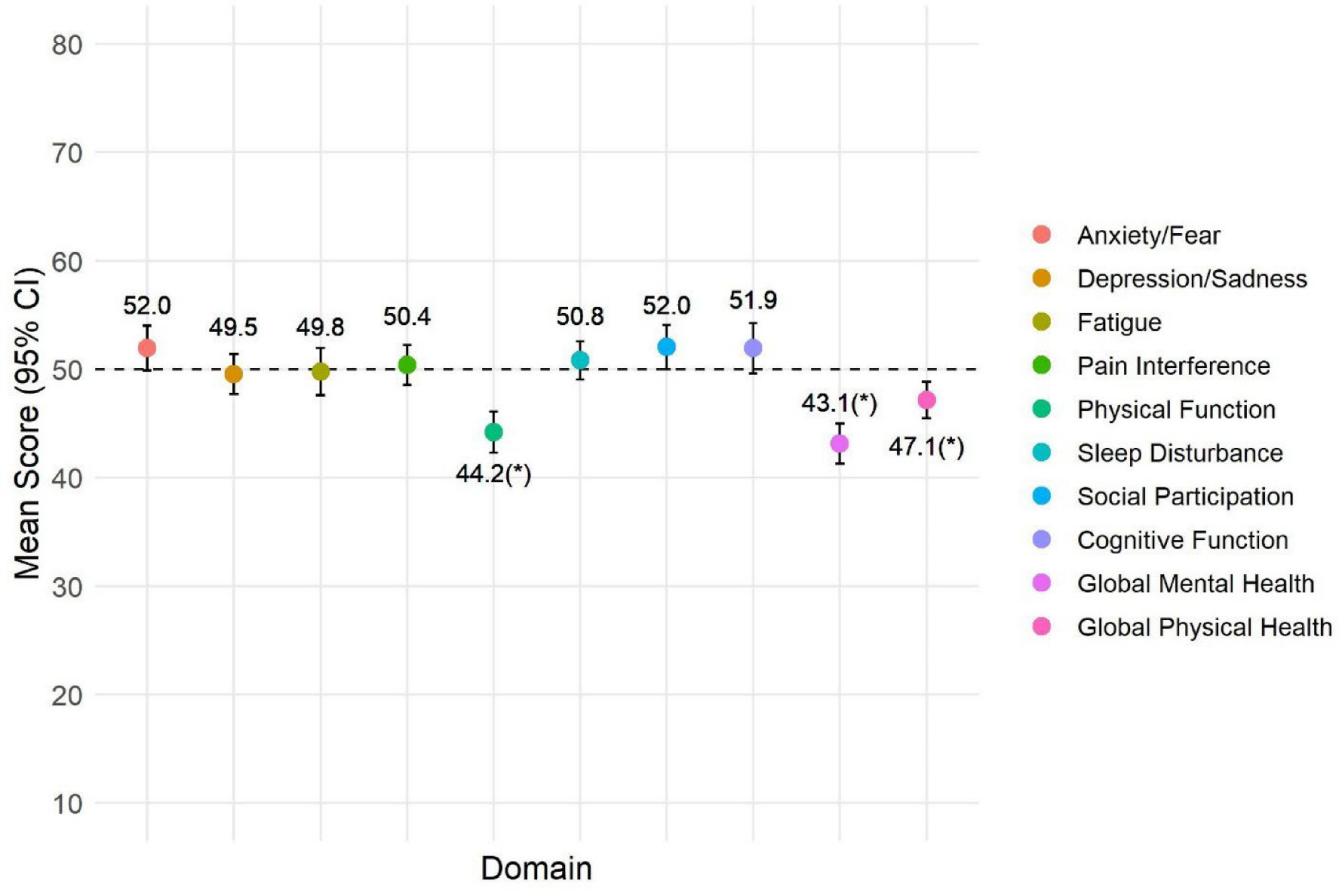
PROMIS-29 scores of survivors of severe COVID-19.

Six months after hospitalization, anxiety, depression, fatigue, sleep disturbance, ability to participate in social roles and activities, pain interference, and cognitive function scores in survivors were not significantly different from the U.S. general population (Figure 2). Physical function (44.2, SD 11.0), however, was significantly worse than that of the population. PROMIS-29 physical and mental health summary scores were 45.2 (SD 11.6) and 50.5 (SD 11.4), respectively. PROMIS Global Physical and Mental Health scores were lower than the U.S. mean of 50 (47.1, SD 9.8 and 43.1, SD 10.8, respectively). Of the 117 patients who answered questions regarding their physical function, cognition, mood, fatigue, sleep, social activities, and pain compared to before COVID-19, the majority of patients felt “about the same” or “better” in all domains except physical function and fatigue, where the majority felt that they were worse (Table E1).

Mean scores of the 7 domains in PROMIS-29 and cognitive function, and global mental and physical health scores of patients who survived severe COVID-19 and returned surveys. Norm-based scores have been calculated for each domain so that a score of 50 (standard deviation [SD] = 10), as indicated by the dotted line, represents the average of the general U.S. population. A score of 40 is one SD lower than the mean of the reference population, while a score of 60 is one SD higher than the mean of the reference population. Higher scores represent worse symptomatology in anxiety, depression, fatigue, sleep disturbance, and pain interference. In physical function, social participation and cognition, a higher score signals better functioning.

*p < 0.05 when compared to a mean of 50.

**Figure 3.**
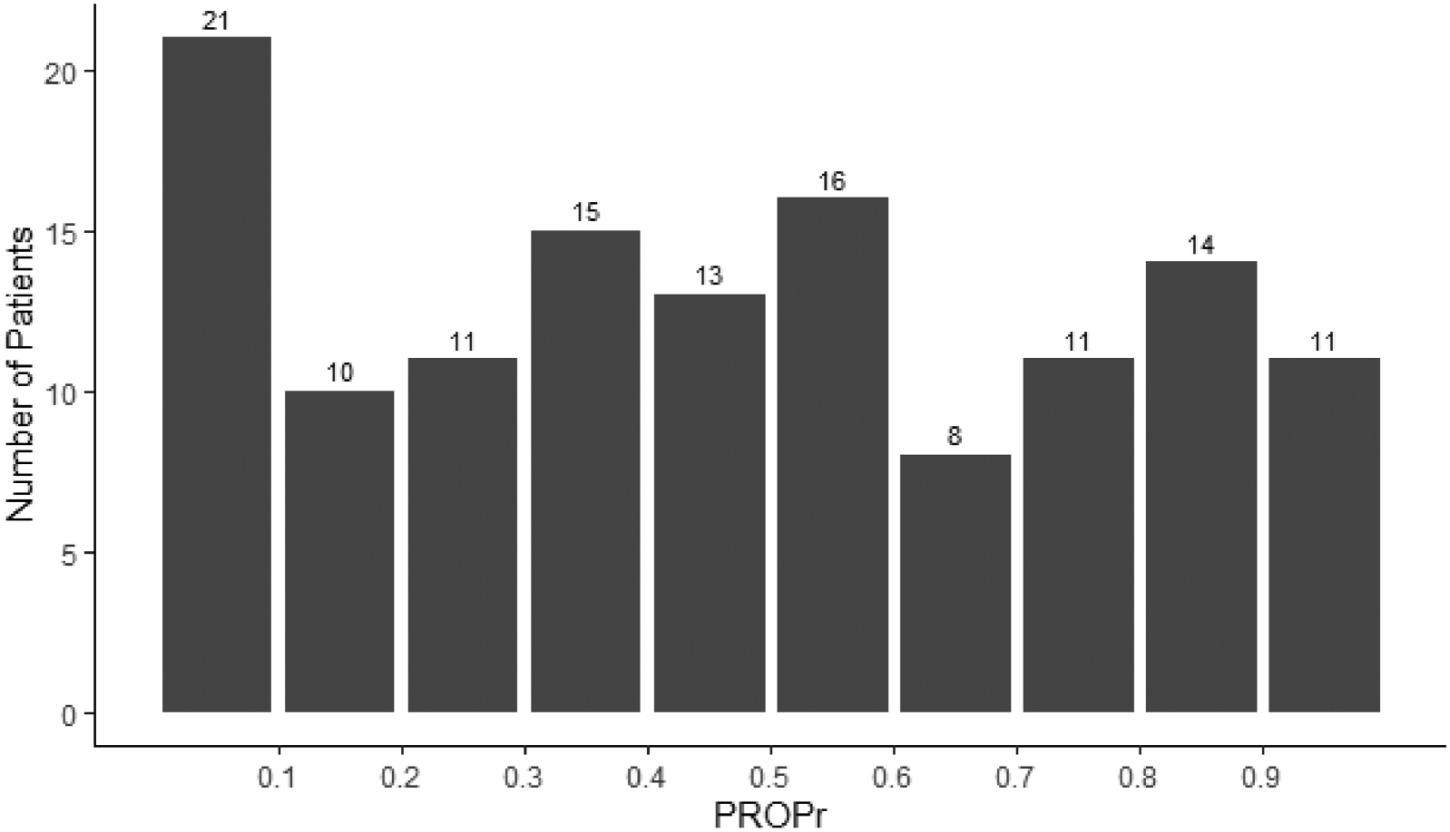
Distribution of PROMIS-preference (PROPr) score.

The mean PROPr preference-based utility score of patients who survived severe COVID-19 was 0.46 (SD 0.30) and ranged from −0.02 to 0.96 (a negative score indicates a state worse than dead and 1 indicates perfect health). In contrast to PROPr scores of a general population in which the mean is 0.52 and approximates a normal distribution,^50^ patients who survived severe COVID-19 had PROPr scores reflecting a relatively uniform (rather than normal) distribution (Figure 3).

PROPr is a preference-based utility score for health states defined by 7 PROMIS domains (depression, fatigue, pain interference, physical function, sleep disturbance, ability to participate in social activities, and cognitive function). It ranges from −0.022 to 1.0 where <0 is worse than dead and 1.0 is perfect health. In a general U.S. population, the distribution of PROPr is nearly a normal distribution.

### Factors Associated with HRQOL Outcome

In bivariate analyses, PROPr was unrelated to demographics or SVI. PROPr was significantly lower for patients with underlying medical conditions (hypertension, diabetes, heart failure, kidney disease and lung disease), transplant, and immunosuppression. Although not associated with ECI or SOFA score at ICU admission, PROPr was associated with mechanical ventilator and vasopressor requirement and a longer hospital length of stay (Table E2).

PROPr was lower in patients who required oxygen or a caregiver at follow up, those readmitted to the hospital, and those who did not return to work or returned to a lower level of employment (Table E2). Nearly a quarter (23.8%) of the ICU survivors had PROPr scores of ≤ 0.2. This group of patients reported fatigue, anxiety, pain, depression and social function more than one standard deviation worse than the general population and physical function nearly two standard deviations worse than the general population average. Nearly 40% required supplemental oxygen, 32% needed a caregiver and 23% had been readmitted to the hospital (Table E3).

### PTSD, Loneliness, and Willingness to Receive ICU Care

The mean sum of the four questions on the PCL-5 was 3.48 (SD 4.33), with 26 (20.3%) having scores ≥ 8, indicating that PTSD is probable (Table 2). The mean score on the 3-item loneliness score was 4.4 (SD 2.0), with 38 (29.2%) patients indicating loneliness (score ≥ 6). Both PTSD and loneliness were significantly negatively correlated with PROPr (Table E3).

Nearly all surveyed, 125 (97.7%), were glad they received aggressive life-sustaining treatment, and 118 (89.4%) would choose to receive it again, if necessary (Table 2). Including the 3 patients who wished they had received comfort care during their severe COVID-19 illness, there were 9 (6.8%) patients who would not want aggressive life-sustaining treatment again if they had recurrent severe COVID-19. Preference for aggressive care was not associated with PROPr (Table E3).

## Discussion

Few studies have evaluated the post-ICU course of adults in the U.S. who were critically ill due to COVID-19. Using both survey and medical record data, we found that survivors of severe COVID-19 had worse physical function, but similar anxiety, depression, fatigue, sleep disturbance, and pain interference scores compared to that of the U.S. general population. Moreover, the mean physical function score was less than one standard deviation below normal. In contrast to other studies,^7-11^ we found evidence for debilitating physical and mental disability in only a small portion of our patient sample. The PROPr score was 0.46 (SD 0.3), which is worse than the mean PROPr score (0.52) observed in the US population;^44,50^ however, this difference is less than the effect of chronic illness on PROPr (ie, emphysema^50^ and dialysis^51^ decrease PROPr by about −0.2). Taking into consideration that the majority of this patient population had at least one baseline co-morbid condition in addition to their risk for PICS, these findings are reassuring. Furthermore, PTSD and loneliness were uncommon, and most patients were able to return home to live independently. Thus, most patients who survive admission to the ICU for severe COVID-19 are in health states 6 months post-discharge that are not very different from that of the general U.S. population.

However, these overall statistics belie the substantial minority of survivors with significant health impacts from COVID-19 following intensive care, reflected by PROPr scores close to zero. Thirty-one patients had health utility scores ≤ 0.2; of which 21 patients had scores less than 0.1 (a score of 0 is comparable to dead). This substantial minority are often tethered to oxygen, newly require a caregiver, and commonly are unable to return to work. Demographic characteristics and social vulnerability do not distinguish this group of patients; compromised outcomes appear to be associated with underlying medical conditions and level of illness during the ICU stay (but not at entry). It is also notable that both Global Physical and Mental Health scores are lower than that of the U.S general population, indicating that the patients’ overall perception of their health is lower than what is indicated by the PROMIS-29 domains. These findings may reflect the more difficult to define symptoms due to “long-COVID”.^52,53^ Identification of factors that can prospectively identify patients who do poorly after severe COVID-19 remains urgently needed.

The overwhelming majority of patients who survived severe COVID were appreciative of the aggressive ICU care they received and would choose to undergo it again. This attitude contrasts starkly with the perspective of intensive care being largely futile commonly displayed in the lay media.^54-56^ Interpretation of this finding should recognize that we were unable to obtain the perspectives of those who had the worst outcomes (ie, those too sick to respond and those who died in the ICU), though nearly half of the non-respondents reported that they were back to baseline health (Figure 1). PROPr, which assigns a score of zero to dead, allows us to provide a global assessment on all patients who required ICU care for COVID-19. Including those who did died along with survey respondents, the overall PROPr is 0.29.

This study has limitations as well as strengths. There was thorough follow-up of patients discharged from the ICU. The measures used in this study have been extensively evaluated and assess multiple dimensions of HRQOL. Limitations include the absence of physiologic pulmonary function assessments or diagnostic imaging, which were deferred given our focus is on patient-reported outcomes. Although demographics, clinical characteristics, and treatments were similar between responders and non-responders, non-responders were sicker according to the ECI and less socially vulnerable. Importantly, we lacked baseline HRQOL data prior to ICU admission, thus limiting our ability to assess changes in function, although we attempted to capture this in retrospective self-assessment in our survey questions, albeit 3 months into the study (eTable 1). Furthermore, our follow up is limited to six-months. Lastly, the single health system source of patients limits generalizability and our COVID-19 ICU survivor cohort was less sick than others in the literature as measured by SOFA, length of ICU stay, and proportion of patients ventilated.^57-59^

In conclusion, patients who survive severe COVID-19 display marked heterogeneity in outcomes six months after hospitalization, with many being in health states that are similar to that of the general US population. However, there exists a substantial minority with severely compromised health status. Mechanisms are needed to identify patients treated for COVID-19 in the ICU who are at risk for severe outcomes.

## Supplemental Material

sj-docx-1-jic-10.1177_08850666221092687 - Supplemental material for Survival After Severe COVID-19: Long-Term Outcomes of Patients Admitted to an Intensive Care Unit Supplemental material, sj-docx-1-jic-10.1177_08850666221092687 for Survival After Severe COVID-19: Long-Term Outcomes of Patients Admitted to an Intensive Care Unit by Thanh H. Neville, Ron D. Hays, Chi-Hong Tseng, Cynthia A. Gonzalez, Lucia Chen, Ashley Hong, Myrtle Yamamoto, Laura Santoso, Alina Kung, Kristin Schwab, Steven Y Chang, Nida Qadir, Tisha Wang and Neil S. Wenger in Journal of Intensive Care Medicine

sj-docx-2-jic-10.1177_08850666221092687 - Supplemental material for Survival After Severe COVID-19: Long-Term Outcomes of Patients Admitted to an Intensive Care Unit Supplemental material, sj-docx-2-jic-10.1177_08850666221092687 for Survival After Severe COVID-19: Long-Term Outcomes of Patients Admitted to an Intensive Care Unit by Thanh H. Neville, Ron D. Hays, Chi-Hong Tseng, Cynthia A. Gonzalez, Lucia Chen, Ashley Hong, Myrtle Yamamoto, Laura Santoso, Alina Kung, Kristin Schwab, Steven Y Chang, Nida Qadir, Tisha Wang and Neil S. Wenger in Journal of Intensive Care Medicine
