# Supplementary material for: Survival After Severe COVID-19: Long-Term Outcomes of Patients Admitted to an Intensive Care Unit
Source: J Intensive Care Med. 2022 Apr 5;37(8):1019–28. doi: 10.1177/08850666221092687 (PMC8990100; doi:10.1177/08850666221092687)
Supplement: sj-docx-1-jic-10.1177_08850666221092687 - Supplemental material for Survival After Severe COVID-19: Long-Term Outcomes of Patients Admitted to an Intensive Care Unit [file sj-docx-1-jic-10.1177_08850666221092687.docx]

**Survival After Severe COVID-19 Survey**

**This survey asks about how you are doing. If the patient is unable to fill in the survey, it can be completed by a family member or caregiver who knows the patient well.**

1. Who is completing the survey?

□ Patient

□ Not the patient 🡺 If you are not the patient, how are you are related? __________________

*Please answer each question from the patient’s perspective.*

**First, we will begin with some questions about how you have been since you left the hospital.**

2. Where are you living now?

□ Home

□ Assisted living facility

□ Skilled Nursing Facility

□ Long term acute care facility

□ Other: ______________________________________________

3. Do you currently use any of the following? *(check all that apply)*

□ Supplemental Oxygen?

🡺 How much? __________ L/min

🡺□ At all times or □ Only on exertion?

🡺 If yes, were you on supplemental oxygen before COVID-19? □ No □ Yes

□ CPAP/BiPAP

🡺 If yes, were you on CPAP/BiPAP before COVID-19? □ No □ Yes

□ Mechanical ventilation

🡺 If yes, were you on a ventilator before COVID-19? □ No □ Yes

□ Tube feeds (tube in the nose or into the stomach)

🡺 If yes, were you on tube feeds before COVID-19? □ No □ Yes

□ TPN (nutrition via vein)

🡺 If yes, were you on TPN before COVID-19? □ No □ Yes

□ Dialysis

🡺 If yes, were you on dialysis before COVID-19? □ No □ Yes

4. If you did not need a caregiver before your COVID-19 illness, do you need one now?

□ I had a caregiver prior to my illness.

□ Yes, I have a paid caregiver now that I didn’t need before

□ Yes, I have an unpaid family member or friend acting as caregiver that I didn’t need before

□ No

Go to next page 🡺

5. Have you been readmitted to the hospital *since* your ICU admission for COVID-19?

□ No

□ Yes 🡺 a) What was the date of that admission? ___________

b) Was it to the ICU? □ No □ Yes

6. Were you were working at a paying job prior to CoVID-19 illness?

□ No 🡺 skip next question

□ Yes

7. If you were working at a paying job prior to your CoVID-19 illness, have you returned to work?

□ No

□ Yes 🡺 How many weeks after hospital discharge did you return to work? ________ weeks

🡺 Are you employed to the level you were before your illness?

□ No □ Yes

**Please respond to each question or statement about your current condition by marking one box per row.**

| **Physical Function** | **Without**  **any difficulty** | **With a**  **little difficulty** | **With**  **some**  **difficulty** | **With**  **much difficulty** | **Unable**  **to do** |
| --- | --- | --- | --- | --- | --- |
| 8. Are you able to do chores such as vacuuming or yard work? | □ | □ | □ | □ | □ |
| 9. Are you able to go up and down stairs at a normal pace? | □ | □ | □ | □ | □ |
| 10. Are you able to go for a walk of at least 15 minutes? | □ | □ | □ | □ | □ |
| 11. Are you able to run errands and shop? | □ | □ | □ | □ | □ |
| 12. Does your health now limit you in taking care of your personal needs (dress, comb hair, toilet, eat, bathe)? | □ | □ | □ | □ | □ |

Go to next page 🡺

| **Thinking**  **In the last 7 days…** | **Never** | **Rarely (one or two times)** | **Sometimes (two or three times)** | **Often (about once per day)** | **Very often (several times a day)** |
| --- | --- | --- | --- | --- | --- |
| 13. My thinking has been slow… | □ | □ | □ | □ | □ |
| 14. It has seemed like my brain was not working as well as usual… | □ | □ | □ | □ | □ |
| 15. I have had to work harder than usual to keep track of what I was doing.. | □ | □ | □ | □ | □ |
| 16. I have had trouble shifting back and forth between different activities that require thinking… | □ | □ | □ | □ | □ |
| 17. I have had trouble concentrating… | □ | □ | □ | □ | □ |
| 18. I have had to work really hard to pay attention or I would make a mistake… | □ | □ | □ | □ | □ |
| 19. I have had trouble forming thoughts…. | □ | □ | □ | □ | □ |
| 20. I have had trouble adding or subtracting…. | □ | □ | □ | □ | □ |

| **Anxiety**  **In the past 7 days….** | **Never** | **Rarely** | **Sometimes** | **Often** | **Always** |
| --- | --- | --- | --- | --- | --- |
| 21. I felt fearful | □ | □ | □ | □ | □ |
| 22. I found it hard to focus on anything other than my anxiety | □ | □ | □ | □ | □ |
| 23. My worries overwhelmed me | □ | □ | □ | □ | □ |
| 24. I felt uneasy | □ | □ | □ | □ | □ |

Go to next page 🡺

| **Depression**  **In the past 7 days….** | **Never** | **Rarely** | **Sometimes** | **Often** | **Always** |
| --- | --- | --- | --- | --- | --- |
| 25. I felt worthless | □ | □ | □ | □ | □ |
| 26. I felt helpless | □ | □ | □ | □ | □ |
| 27. I felt depressed | □ | □ | □ | □ | □ |
| 28. I felt hopeless | □ | □ | □ | □ | □ |

| **Fatigue**  **During the past 7 days…** | **Not at all** | **A little bit** | **Somewhat** | **Quite a bit** | **Very much** |
| --- | --- | --- | --- | --- | --- |
| 29. I felt fatigued | □ | □ | □ | □ | □ |
| 30. I have trouble starting things because I am tired | □ | □ | □ | □ | □ |
| 31. How run-down did you feel on average? | □ | □ | □ | □ | □ |
| 32. How fatigued were you on average? | □ | □ | □ | □ | □ |

| **Sleep Disturbance**  **In the past 7 days…** | **Very poor** | **Poor** | **Fair** | **Good** | **Very good** |
| --- | --- | --- | --- | --- | --- |
| 33. My sleep quality was…. | □ | □ | □ | □ | □ |
| In the last 7 days…. | **Not at all** | **A little bit** | **Somewhat** | **Quite a bit** | **Very much** |
| 34. My sleep was refreshing | □ | □ | □ | □ | □ |
| 35. I had a problem with sleep | □ | □ | □ | □ | □ |
| 36. I had difficulty falling asleep | □ | □ | □ | □ | □ |

Go to next page 🡺

| **Social Roles and Activities** | **Never** | **Rarely** | **Sometimes** | **Often** | **Always** |
| --- | --- | --- | --- | --- | --- |
| 37. I have trouble doing all of my regular leisure activities with others | □ | □ | □ | □ | □ |
| 38. I have trouble doing all of the family activities that I want to do | □ | □ | □ | □ | □ |
| 39. I have trouble doing all of my usual work (include work at home) | □ | □ | □ | □ | □ |
| 40. I have trouble doing all of the activities with friends that I want to do | □ | □ | □ | □ | □ |

| **Pain Interference**  **In the past 7 days…** | **Not at all** | **A little bit** | **Somewhat** | **Quite a bit** | **Very much** |
| --- | --- | --- | --- | --- | --- |
| 41. How much did pain interfere with your day to day activities? | □ | □ | □ | □ | □ |
| 42. How much did pain interfere with work around the home? | □ | □ | □ | □ | □ |
| 43. How much did pain interfere with your ability to participate in social activities? | □ | □ | □ | □ | □ |
| 44. How much did pain interfere with your household chores? | □ | □ | □ | □ | □ |

| **Pain Intensity**  **In the past 7 days…**  45. How would you rate your pain on average? | □  0  No pain | □  1 | □  2 | □ 3 | □  4 | □  5 | □  6 | □  7 | □  8 | □  9 | □  10  Worst pain imaginable |
| --- | --- | --- | --- | --- | --- | --- | --- | --- | --- | --- | --- |

Go to next page 🡺

|  | **Excellent** | **Very good** | **Good** | **Fair** | **Poor** |
| --- | --- | --- | --- | --- | --- |
| 46. In general, would you say your quality of life is: | □ | □ | □ | □ | □ |
| 47. In general, how would you rate your physical health? | □ | □ | □ | □ | □ |
| 48. In general, how would you rate your mental health, including your mood and your ability to think? | □ | □ | □ | □ | □ |

**Now, we would like to know how you are *now* compared to *before* you were hospitalized with COVID-19.**

| In general… | **Much better** | **A little better** | **About the same** | **A little worse** | **Much worse** |
| --- | --- | --- | --- | --- | --- |
| 49. How is your physical functioning now compared to before you had COVID-19? | □ | □ | □ | □ | □ |
| 50. How is your thinking (also known as cognition) now compared to before you had COVID-19? | □ | □ | □ | □ | □ |
| 51. How is your mood now compared to before you had COVID-19? | □ | □ | □ | □ | □ |
| 52. How is your fatigue now compared to before you had COVID-19? | □ | □ | □ | □ | □ |
| 53. How is your sleep now compared to before you had COVID-19? | □ | □ | □ | □ | □ |
| 54. How is your ability to participate in social roles and activities now compared to before you had COVID-19? | □ | □ | □ | □ | □ |
| 55. How is your pain now compared to before you had COVID-19? | □ | □ | □ | □ | □ |

Go to next page 🡺

**Please read each sentence and select one answer indicating how much you have been bothered by the COVID-19 ICU experience *in the past month.***

| **In the past month…** | **Not at all** | **A little bit** | **Moderately** | **Quite a bit** | **Extremely** |
| --- | --- | --- | --- | --- | --- |
| 56. Suddenly feeling or acting as if the ICU experience were happening again (as if you were actually back there reliving it)? | □ | □ | □ | □ | □ |
| 57. Avoiding external reminders of the ICU experience (for example, people, places, conversations, activities, objects, or situations)? | □ | □ | □ | □ | □ |
| 58. Feeling distant or cut off from other people? | □ | □ | □ | □ | □ |
| 59. Irritable behavior, angry outbursts, or acting aggressively? | □ | □ | □ | □ | □ |

**The next 3 questions ask about how you have felt since leaving the hospital.**

**Since your hospitalization for COVID-19**…

|  | **Hardly ever** | **Some of the time** | **Often** |
| --- | --- | --- | --- |
| 60. How often do you feel that you lacked companionship? | □ | □ | □ |
| 61. How often do you feel left out? | □ | □ | □ |
| 62. How often do you feel isolated? | □ | □ | □ |

Go to next page 🡺

**The next few questions ask about how COVID-19 has affected you financially.**

|  | **Not at all** | **A little bit** | **Somewhat** | **Quite a bit** | **Very much** |
| --- | --- | --- | --- | --- | --- |
| 63. I worry about the financial problems I will have in the future as a result of my COVID-19 illness | □ | □ | □ | □ | □ |
| 64. I am frustrated that I cannot work or contribute as much as I usually do | □ | □ | □ | □ | □ |
| 65. COVID-19 has reduced my satisfaction with my present financial situation | □ | □ | □ | □ | □ |
| 66.I feel in control of my financial situation | □ | □ | □ | □ | □ |

**Now we ask you to think back to your hospitalization that included care in the ICU.**

67. Are you glad that you received aggressive life-sustaining treatment for COVID-19 when you were in the hospital?

□ No, I would rather have had comfort care even though I probably would have died

□ Yes, I am glad I received life-sustaining treatment

If yes, would you be willing to receive this type of treatment again, if needed?

□ No □ Yes

Go to next page 🡺

**Lastly, we’d like to know a little more about you and your household.**

68. What is your highest level of education? *(Choose one)*

□ Less than high school graduate

□ High school graduate or GED equivalent

□ Some college

□ College graduate

□ Post graduate education

69. What is your total annual household income? *(Choose one)*

□ Less than $20,000

□ $20,000 to $34,999

□ $35,000 to $49,999

□ $50,000 to $74,999

□ $75,000 to $99,999

□ $100,000 to $150,000

□ More than $150,000

70. What is the total number of people who live in your home currently? (include yourself)

□ 1

□ 2

□ 3

□ 4

□ 5

□ More than 5

71. Is there anything else you’d like to tell us about how you are doing or your “after COVID-19” experience?

_________________________________________________________________________________________________________________________________________________________________________________________________________________________________________________________________________________________________________________________________________________________

**THANK YOU! YOU ARE DONE!**

To receive your $25 Amazon gift card, please mail back this completed survey in the pre-paid envelope. Please write the email address you’d like for the gift-card code to be emailed to:

__________________________________________________________________________

□ If you would like the card mailed to your home instead of e-mailed, please check this box.
