# Supplementary material for: Survival After Severe COVID-19: Long-Term Outcomes of Patients Admitted to an Intensive Care Unit
Source: J Intensive Care Med. 2022 Apr 5;37(8):1019–28. doi: 10.1177/08850666221092687 (PMC8990100; doi:10.1177/08850666221092687)
Supplement: sj-docx-2-jic-10.1177_08850666221092687 - Supplemental material for Survival After Severe COVID-19: Long-Term Outcomes of Patients Admitted to an Intensive Care Unit [file sj-docx-2-jic-10.1177_08850666221092687.docx]

Supplement tables

Table E1. Patients’ retrospective comparison of their current condition to before COVID-19

|  | **Much better** | **A little better** | **About the same** | **A little worse** | **Much worse** |
| --- | --- | --- | --- | --- | --- |
| How is your physical functioning now compared to before you had COVID-19? | 9 (7.9%) | 8 (7.0%) | 35 (30.7%) | 38 (33.3%) | 24 (21.1%) |
| How is your thinking (also known as cognition) now compared to before you had COVID-19? | 8 (7.1%) | 5 (4.4%) | 60 (53.1%) | 27 (23.9%) | 13 (11.5%) |
| How is your mood now compared to before you had COVID-19? | 8 (7.1%) | 11 (9.7%) | 54 (47.8%) | 25 (22.1%) | 15 (13.3%) |
| How is your fatigue now compared to before you had COVID-19? | 6 (5.3%) | 9 (7.9%) | 36 (31.6%) | 39 (34.2%) | 24 (21.1%) |
| How is your sleep now compared to before you had COVID-19? | 6 (5.3%) | 8 (7.0%) | 47 (41.2%) | 34 (29.8%) | 19 (16.7%) |
| How is your ability to participate in social roles and activities now compared to before you had COVID-19? | 7 (6.1%) | 6 (5.3%) | 50 (43.9%) | 32 (28.1%) | 19 (16.7%) |
| How is your pain now compared to before you had COVID-19? | 12 (11.1%) | 4 (3.7%) | 55 (50.9%) | 22 (20.4%) | 15 (13.9%) |

Table E2. Relationship of PROPr with Patient Characteristics (n=130)

|  | PROPr, median (IQR) | Correlation with PROPr | p-value |
| --- | --- | --- | --- |
| DEMOGRAPHIC FACTORS | | | |
| Age |  | -0.09 | 0.30 |
| Race  White Non-Hispanic (N=23)  Hispanic (N=78)  Black (N=9)  Asian (N=11)  Other (N=9) | 0.5 (0.3-0.6)  0.5 (0.2-0.8)  0.2 (0.1-0.5)  0.5 (0.4-0.6)  0.3 (0.2-0.5) |  | 0.55 |
| Gender  Female (N=59)  Male (N=71) | 0.5 (0.3-0.8)  0.5 (0.1-0.7) |  | 0.15 |
| Language  English (N=84)  Spanish (N=38)  Other (N=8) | 0.4 (0.2-0.7)  0.6 (0.3-0.8)  0.4 (0.3-0.6) |  | 0.17 |
| Insurance  Private (N=59)  Public/Medicaid (N=62)  Other/Unknown (N=9) | 0.4 (0.2-0.7)  0.5 (0.2-0.8)  0.5 (0.2-0.6) |  | 0.57 |
| Social Vulnerability Index, total |  | 0.02 | 0.83 |
| CLINICAL FACTORS | | | |
| Hypertension  Yes (N=70)  No (N=60) | **0.4 (0.2-0.6)**  **0.6 (0.2-0.8)** |  | 0.040 |
| Diabetes  Yes (N=50)  No (N=80) | 0.5 (0.2-0.7)  0.4 (0.2-0.8) |  | 0.93 |
| Heart failure  Yes (N=10)  No (N=120) | **0.2 (0-0.5)**  **0.5 (0.2-0.8)** |  | 0.019 |
| Chronic kidney disease  Yes (N=21)  No (N=109) | **0.3 (0.1-0.6)**  **0.5 (0.2-0.8)** |  | 0.045 |
| Chronic pulmonary disease  Yes (N=15)  No (N=115) | **0.2 (0.1-0.4)**  **0.5 (0.2-0.8)** |  | 0.015 |
| Organ transplant  Yes (N=13)  No (N=117) | 0.4 (0.2-0.6)  0.5 (0.2-0.7) |  | 0.52 |
| Immunosuppressed (not due to transplant)  Yes (N=10)  No (N=119) | **0.3 (0.1-0.4)**  **0.5 (0.2-0.8)** |  | 0.039 |
| Body Mass Index |  | -0.10 | 0.24 |
| Elixhauser Index |  | -0.13 | 0.13 |
| SOFA on ICU Admission |  | -0.12 | 0.17 |
| Hospital length of stay, days |  | **-0.24** | 0.005 |
| ICU length of stay |  | -0.17 | 0.061 |
| Mechanical Ventilation  Yes (N=48)  No (N=82) | **0.3 (0.1-0.6)**  **0.5 (0.3-0.8)** |  | 0.018 |
| Vasopressors  Yes (N=48)  No (N=82) | **0.3 (0.1-0.6)**  **0.5 (0.3-0.8)** |  | 0.005 |
| Hemodialysis  Yes (N=9)  No (N=121) | 0.4 (0.2-0.7)  0.5 (0.2-0.7) |  | 0.78 |
| Extracorporeal Membrane Oxygenation  Yes (N=5)  No (N=125) | 0.5 (0.2-0.5)  0.5 (0.2-0.7) |  | 0.68 |
| OUTCOMES | | | |
| Requires oxygen at follow up*  Yes (N=22)  No (N=108) | **0.2 (0-0.4)**  **0.5 (0.3-0.8)** |  | <0.001 |
| Requires caregiver at follow up*  Yes (N=42)  No (N=98) | **0.2 (0-0.4)**  **0.6 (0.3-0.8)** |  | <0.001 |
| Readmitted to hospital  Yes (N=12)  No (N=117) | **0.1 (0-0.3)**  **0.5 (0.2-0.8)** |  | <0.001 |
| Returned to work if worked at baseline  Yes (N=40)  No (N=28) | **0.7 (0.3-0.8)**  **0.4 (0.2-0.6)** |  | 0.015 |
| Employed at prior level if returned to work  Yes (N=32)  No (N=6) | **0.8 (0.5-0.9)**  **0.2 (0-0.3)** |  | 0.002 |

ICU = intensive care unit, IQR = interquartile range, PROPr = Patient-Reported Outcomes Measurement Information System (PROMIS) Preference summary scores, SOFA = Sequential Organ Failure Assessment,

*Two of the patients required oxygen prior to COVID-19 and 12 had a caregiver prior to COVID-19 hospital admission.

**Bold** values p<0.05. Kruskal-Wallis tests were used to test the association between PROPr and categorical variables, and Pearson’s correlation coefficient for continuous variables.

Table E3 Characteristics of Patients With PROPr scores > 0.2 and ≤ 0.2 (N=130)

|  | Patients with PROPr > 0.2  (N =99) | Patients with PROPr ≤ 0.2  (N=31) | p-value |
| --- | --- | --- | --- |
| Age, years (IQR) | 59.0 (47.9-67.4) | 61.0 (46.8-75.4) | 0.39 |
| Race, N (%)  Non-Hispanic White  Hispanic  Black  Asian  Other | 18 (18.2%)  58 (58.6%)  6 (6.1%)  10 (10.1%)  7 (7.1%) | 5 (16.1%)  20 (64.5%)  3 (9.7%)  1 (3.2%)  2 (6.5%) | 0.77 |
| Female, N (%) | 41 (41.4%) | 18 (58.1%) | 0.15 |
| Insurance, N (%)  Private  Public/Medical/Medicaid  Other/Unknown | 45 (45.5%)  48 (48.5%)  6 (6.1%) | 14 (45.2%)  14 (45.2%)  3 (9.7%) | 0.73 |
| SVI, total (IQR) | 0.7 (0.3-0.8) | 0.6 (0.3-0.8) | 0.83 |
| Underlying Conditions, N (%)  Hypertension  Diabetes  Heart failure  Chronic kidney disease  Chronic lung disease  Organ transplant recipient  Immunosuppressed (not due to transplant) | 53 (53.5%)  39 (39.4%)  5 (5.1%)  15 (15.2%)  9 (9.1%)  10 (10.1%)  6 (6.1%) | 17 (54.8%)  11 (35.5%)  5 (16.1%)  6 (19.4%)  6 (19.4%)  3 (9.7%)  4 (12.9%) | 1.0  0.86  0.06  0.78  0.9  1.0  0.67 |
| Elixhauser Index (IQR) | 8.0 (0.0-16.0) | 10.0 (0.0-18.5) | 0.46 |
| SOFA on ICU Admission (IQR) | 3.0 (0.0-5.0) | 4.0 (0.5-8.0) | 0.17 |
| Hospital length of stay, days (IQR) | 13.0 (8.0-18.0) | 20.0 (9.0-29.0) | 0.05 |
| Utilized during hospital stay, N (%):  Mechanical Ventilation  Vasopressors  Hemodialysis  Extracorporeal Membrane Oxygenation | 32 (32.3%)  **31 (31.3%)**  7 (7.1%)  3 (3.0%) | 16 (51.6%)  **17 (54.8%)**  2 (6.5%)  2 (6.5%) | 0.08  0.03  1.0  0.59 |
| Currently utilizing oxygen, N (%) | **10 (10.1%)** | **12 (38.7%)** | <0.001 |
| Currently needs a caregiver, N (%) | **12 (12%)** | **10 (32%)** | <0.001 |
| Readmitted to hospital, N (%) | **5 (5.1%)** | **7 (22.6%)** | 0.01 |
| If working before, returned to work, N (%) | 35 (61.4%) | 5 (45.5%) | 0.34 |
| Employed to level prior to admission, N (%) | **30 (90.9%)** | **2 (40.0%)** | 0.02 |
| PTSD Checklist-5, N (%) with score ≥ 8 | **8 (8.3%)** | **17 (56.7%)** | <0.001 |
| Loneliness Scale, N (%) with score | **13 (13.4%)** | **24 (77.4%)** | <0.001 |
| Glad received aggressive life-sustaining treatment for COVID-19  No  Yes | 2 (2.1%)  93 (97.9%) | 1 (3.2%)  30 (96.8%) | 1 |
| Willing to receive aggressive life-sustaining treatment for COVID-19 again, if needed  No  Yes | 4 (4.3%)  88 (95.7%) | 2 (6.7%)  28 (93.3%) | 0.64 |
| PROMIS domain score, mean (SD)  Anxiety/Fear  Cognitive Function  Depression/Sadness  Fatigue  Pain Interference  Physical Function  Sleep Disturbance  Social Participation | **48.0 (9.1)**  **56.7 (8.7)**  **45.8 (7.3)**  **45.1 (9.6)**  **46.3 (7.0)**  **48.2 (8.7)**  **48.1 (8.7)**  **56.9 (8.3)** | **64.9 (10.5)**  **36.7 (13.9)**  **61.6 (10.8)**  **64.4 (9.7)**  **62.9 (10.3)**  **31.3 (6.8)**  **59.7 (9.1)**  **36.6 (7.8)** | <0.001  <0.001  <0.001  <0.001  <0.001  <0.001  <0.001  <0.001 |

ICU = intensive care unit, IQR = interquartile range, SD = standard deviation, PROPr = PROMIS-Preference summary scores, SVI = social vulnerability index, SOFA = Sequential Organ Failure Assessment, PROMIS = Patient-Reported Outcomes Measurement Information System (where score is 50 (SD=10) in general U.S. population

**Bold** values p<0.05. χ2, Fisher’s exact, two-sample t-tests, and Wilcoxon rank-sum tests used as appropriate.
